# Distinct anti-oncogenic effect of various microRNAs in different mouse models of liver cancer

**DOI:** 10.18632/oncotarget.3166

**Published:** 2015-02-09

**Authors:** Junyan Tao, Junfang Ji, Xiaolei Li, Ning Ding, Heng Wu, Yan Liu, XinWei Wang, Diego F. Calvisi, Guisheng Song, Xin Chen

**Affiliations:** ^1^ School of Pharmacy, Hubei University of Chinese Medicine, Wuhan, Hubei, P.R. China; ^2^ Department of Bioengineering and Therapeutic Sciences and Liver Center, University of California, San Francisco, California, USA; ^3^ Liver Carcinogenesis Section, Laboratory of Human Carcinogenesis, Center for Cancer Research, National Cancer Institute, Bethesda, Maryland, USA; ^4^ Cancer Biology Program, University of Hawaii Cancer Center, University of Hawaii at Manoa, Honolulu, Hawaii, USA; ^5^ Department of Hepatobiliary Surgery, Xijing Hospital, The Fourth Military Medical University, Xi’an, Shaanxi, P.R. China; ^6^ Key Laboratory of Carcinogenesis and Translational Research (Ministry of Education), Department of Gastrointestinal Surgery, Peking University Cancer Hospital and Institute, Beijing, P.R. China; ^7^ Department of Medicine, University of Minnesota Medical School, Minneapolis, Minnesota, USA; ^8^ Institute of Pathology, University of Greifswald, Greifswald, Germany

**Keywords:** HCC, c-Myc, AKT, Ras, mouse liver cancer

## Abstract

Deregulation of microRNAs (miRNAs) is a typical feature of human hepatocellular carcinoma (HCC). However, the *in vivo* relevance of miRNAs along hepatocarcinogenesis remains largely unknown. Here, we show that liver tumors induced in mice by c-Myc overexpression or AKT/Ras co-expression exhibit distinct miRNA expression profiles. Among the downregulated miRNAs, eight (miR-101, miR-107, miR-122, miR-29, miR-365, miR-375, miR-378, and miR-802) were selected and their tumor suppressor activity was determined by overexpressing each of them together with c-Myc or AKT/Ras oncogenes in mouse livers via hydrodynamic transfection. The tumor suppressor activity of these microRNAs was extremely heterogeneous in c-Myc and AKT/Ras mice: while miR-378 had no tumor suppressor activity, miR-107, mir-122, miR-29, miR-365 and miR-802 exhibited weak to moderate tumor suppressor potential. Noticeably, miR-375 showed limited antineoplastic activity against c-Myc driven tumorigenesis, whereas it strongly inhibited AKT/Ras induced hepatocarcinogenesis. Furthermore, miR-101 significantly suppressed both c-Myc and AKT/Ras liver tumor development. Altogether, the present data demonstrate that different oncogenes induce distinct miRNA patterns, whose modulation differently affects hepatocarcinogenesis depending on the driving oncogenes. Finally, our findings support a strong tumor suppressor activity of miR-101 in liver cancer models regardless of the driver oncogenes involved, thus representing a promising therapeutic target in human HCC.

## INTRODUCTION

Hepatocellular carcinoma (HCC), the most frequent primary tumor of the liver, is one of the leading causes of cancer-related death worldwide [1]. In the U.S., the incidence of HCC has been on the rise during the past decade [2]. According to the American Cancer Society, there will be 30,640 newly diagnosed cases and 21,670 deaths due to HCC in the US in the year 2014 (www.cancer.org). Treatment options for HCC are very limited [3, 4]. Surgical resection and liver transplantation are the only curative therapeutic approaches, but they apply only to early stage tumors, and liver transplantation is limited by the availability of liver donors [5]. Sorafenib, a multi-kinase inhibitor targeting the Raf/VEGFR/PDGFR/c-Kit axis, is the only FDA approved drug for HCC treatment [6, 7]. However, it has very limited efficacy in improving the length of patients’ survival [8]. Thus, the identification of novel therapeutic targets for the treatment of this malignancy is mandatory. One of the major challenges in drug development for HCC treatment resides in the fact that HCC is a highly heterogeneous disease at the molecular level, therefore, it is conceivable that only certain HCC subsets would benefit of the use of targeted therapies.

MicroRNAs (miRNAs) are evolutionally conserved non-coding small RNAs that regulate the transcription and translation of genes. miRNAs predominantly bind to the 3′ end of UTR region of target mRNAs, leading to degradation of mRNAs or inhibition of the mRNA translation. miRNAs have been demonstrated as key regulators of carcinogenesis, acting as *bona fide* oncogenes or tumor suppressor genes depending on the cellular functions of their target genes. A large number of miRNAs whose expression is deregulated in human HCC specimens has been identified [9–11]. Subsequent functional studies further supported their critical role(s) in hepatocarcinogenesis by their ability to modulate cell proliferation, survival, and invasive properties. Based on this body of evidence, it can be envisaged that some miRNAs represent novel, potential targets for HCC treatment. One of the major advantages of modulating miRNAs for therapeutic purposes consists on the fact that targeting one miRNA simultaneously affects multiple genes and signaling cascades. Thus, it can be hypothesized that modulation of a single miRNAs might regulate the growth of human HCCs with heterogeneous molecular features. In accordance with the latter hypothesis, a number of studies performed either *in vitro* or in xenograft models have shown that modulating miRNA expression in HCCs has a significant therapeutic potential [12–15]. Despite these promising results, delivery of miRNAs into cells has been the major challenge for miRNA based therapeutics so far [16]. Nonetheless, liver has been shown to be the primary site in the body for the uptake of miRNAs molecules [17]. Thus, it seems likely that miRNAs can be delivered to HCC cells, and miRNA based target therapy might be beneficial for liver cancer patients [17]. Despite this body of evidence, many information regarding miRNAs and their regulation along hepatocarcinogenesis are missing. In addition, it remains unclear which specific miRNAs are able to successfully inhibit an oncogenic stimulus induced by a certain gene.

Amplification and overexpression of the c-Myc oncogene is a frequent event in human HCC [18, 19]. In mice, overexpression of c-Myc via hydrodynamic transfection leads to rapid liver tumor formation [20]. In addition, coordinated activation of AKT/mTOR and Ras/MAPK cascades occurs in over 50% of all human HCCs, and is associated with biological aggressiveness and poor prognosis. This phenotype can be recapitulated *in vivo* by hydrodynamically transfecting activated forms of AKT (myr-AKT) and NRas (NRas-V12) oncogenes (AKT/Ras) into the mouse liver [21].

In the present investigation, we determined the miRNA expression profiles in liver tumors from c-Myc and AKT/Ras mice. We found that miRNA profiles were distinct between c-Myc and AKT/Ras liver tumors. Eight miRNAs (miR-101, miR-107, miR-122, miR-29, miR-365, miR-375, miR-378, and miR-802), whose expression was found to be downregulated in c-Myc and/or AKT/Ras liver tumors, were selected and their tumor suppressor activity was assessed in c-Myc and AKT/Ras mice. The results from this study indicate that the selected miRNAs possess a heterogeneous tumor suppressor activity *in vivo*. Importantly, we found that miR-101 strongly inhibited both c-Myc and AKT/Ras induced hepatocarcinogenesis. Overexpression of miR-101 in the liver might represent a novel and efficient strategy for HCC prevention and treatment.

## RESULTS

### Distinct miRNA expression profiles characterize c-Myc and AKT/Ras induced liver tumors

To characterize miRNAs regulated by different oncogene stimuli during hepatocarcinogenesis, we compared the global miRNA expression patterns of normal mouse livers with those from liver tumors induced by c-Myc or AKT/Ras oncogenes (*n* = 9 total; and *n* = 3 in each group). A total of 599 mouse miRNAs were analyzed (Supplementary Table 1A).

**Figure 1:**
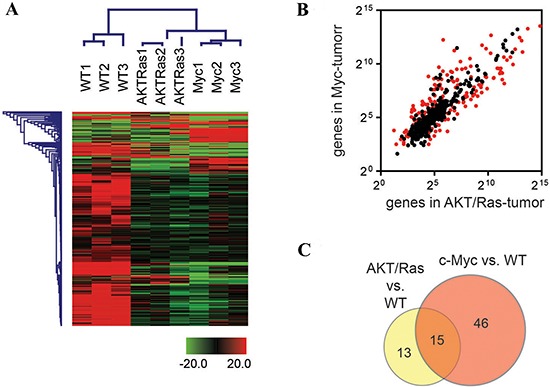
c-Myc and AKT/Ras induced liver tumors exhibit distinct miRNA profiles **(A)** Unsupervised hierarchical clustering analysis using 599 mouse miRNAs in normal livers from wild-type (WT) mice and liver tumors from c-Myc and AKT/Ras injected mice. **(B)** Scattering Plot of miRNAs in liver tumors from c-Myc and AKT/Ras injected mice. Red spots refer to miRNAs with significant difference (unpaired *t*-test, *p* < 0.05) between c-Myc and AKT/Ras injected mice. **(C)** Venn Diagram analysis of c-Myc signature and AKT/Ras signature. The signature was obtained from the miRNA profiling comparison of c-Myc-tumor (or AKT/Ras tumor) and non-tumor liver from wild-type mice. Unpaired *t*-test was used and miRNAs with *p*-value < 0.001 and fold change ≥ 2 or ≤ 0.5 were included in the signature.

Using unsupervised clustering analysis, miRNA profiles showed a marked difference in the overall microRNA transcriptional pattern between normal livers and liver tumors from AKT/Ras and c-Myc injected mice as well as between AKT/Ras- and c-Myc-driven tumors (Figure 1A). In particular, 27.4% of miRNAs (164 out of 599) were differentially expressed (*p* < 0.05, *t*-test) between AKT/Ras and c-Myc tumors (Figure 1B). Furthermore, 28 miRNAs were significantly differently expressed (*t*-test, *p* < 0.001, > 2-fold difference) between AKT/Ras tumors and normal liver tissues, and were indicated as AKT/Ras liver tumor signature (Supplementary Table 1B). In addition, 61 miRNAs were significantly different (*t*-test, *p* < 0.001, > 2-fold difference) between c-Myc tumors and normal liver tissues (Figure 1C), and were designated as c-Myc liver tumor signature (Supplementary Table 1C). Importantly, AKT/Ras and c-Myc tumors shared only a small number of miRNAs (Figure 1C), clearly indicating the presence of distinct miRNA signatures in tumors induced by different oncogenes.

**Figure 2:**
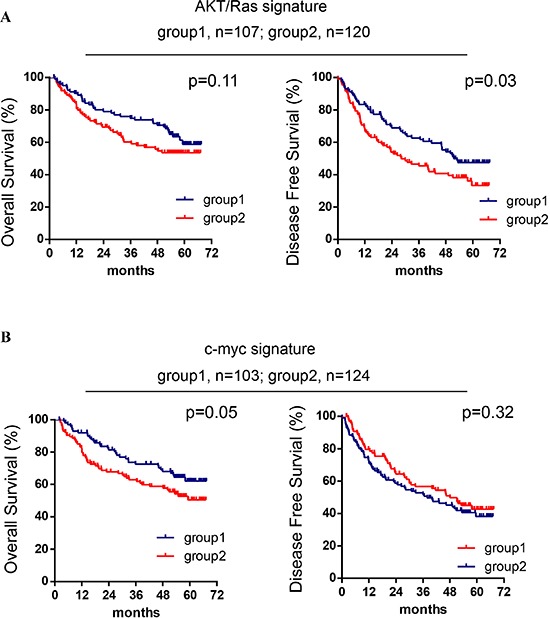
AKT/Ras and c-Myc mouse liver tumor signatures are related to patients’ HCC prognosis **(A)** Kaplan-Meier analysis of overall survival and disease-free survival in HCC cases (*n* = 227) based on the classification of group 1 and group 2 by the AKT/Ras signature. **(B)** Kaplan-Meier analysis of overall survival and disease-free survival in HCC cases (*n* = 227) based on the classification of group 1 and group 2 by the c-Myc signature. Cox-Mantel log-rank test was performed.

To further examine whether AKT/Ras and c-Myc signatures are related to human HCC patients’ prognosis, we performed the integration analysis of these two mouse liver tumor signatures with a published microRNA microarray database of a HCC cohort (*n* = 241) [22–24]. We found that nine of 28 AKT/Ras-related microRNAs were represented in the human miRNA array data. In particular, 35 probes referring to these 9 miRNAs were able to sub-classify human HCC cases in two groups with different disease-free survival (Figure 2A). Furthermore, 161 probes representing 40 c-Myc-related miRNAs were able to separate human HCC cases in two groups with different overall survival (Figure 2B).

### Lack tumor suppressor activity of miR-378 on c-Myc and AKT/Ras induced hepatocarcinogenesis

**Figure 3:**
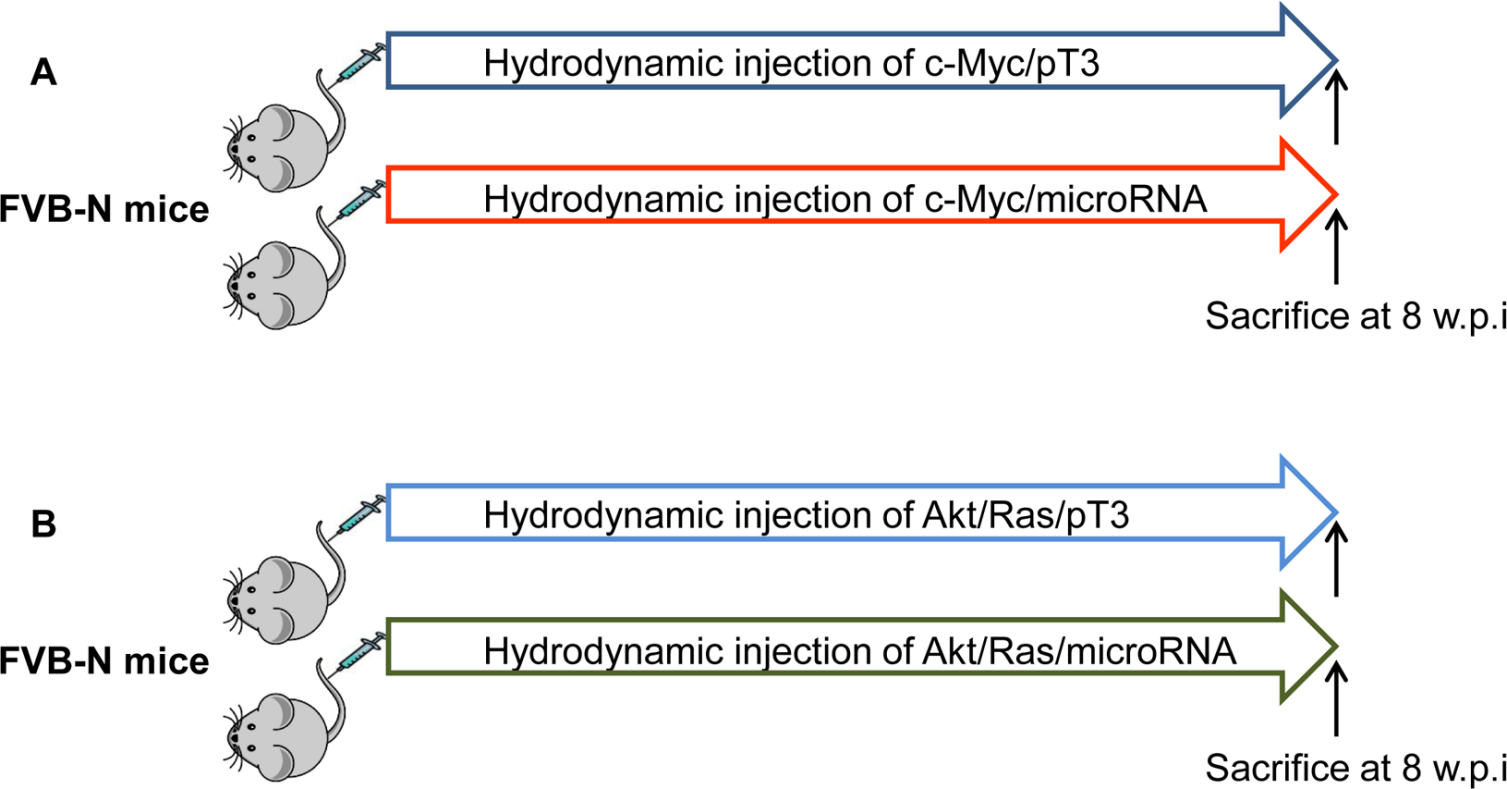
Overall diagram of study design to investigate tumor suppressor activity of miRNA in mice Each of the eight miRNAs was co-expressed with c-Myc **(A)** or AKT/Ras **(B)** oncogenes in the mouse liver via hydrodynamic transfection. pT3-EF1α empty vector was also co-injected with c-Myc (c-Myc/pT3) or AKT/Ras (AKT/Ras/pT3) as a control. w.p.i: weeks post injection.

Using c-Myc and AKT/Ras induced liver tumor as a model system, we determined the tumor suppressor activity of selected miRNAs *in vivo*. The miRNAs were chosen based on the following criteria: first, miRNAs downregulated in c-Myc and/or AKT/Ras tumors were selected. Second, we searched the literature for miRNAs that were reported to be significantly downregulated in human HCC samples, and have been shown to have tumor suppressor potential in liver or other types of tumor cells. Based on the results of our search, a total of 8 miRNAs were investigated: miR-101, miR-107, miR-122, miR-29, miR-365, miR-375, miR-378, and miR-802. Among the 8 miRNAs, 4 miRNA (miR-101, miR-29, miR-107 and miR-122) had available human miRNA array data. We found that the expression of all 4 miRNAs in tumors was significantly related to patient outcomes (Supplementary Figure 1), whereas their levels in non-tumor liver tissues were not (data not shown). As a first step of our tumor suppressor miRNA investigation, we validated the expression of these 8 miRNAs using qRT-PCR. The 8 miRNAs were indeed downregulated in c-Myc and AKT/Ras liver tumor samples (Supplementary Figure 2). To test the hypothesis that overexpression of these miRNAs might possess tumor suppressor activity *in vivo*, each of the eight miRNAs was co-expressed with c-Myc or AKT/Ras oncogenes in the mouse liver via hydrodynamic transfection. pT3-EF1α empty vector was also co-injected with c-Myc (c-Myc/pT3) or AKT/Ras (AKT/Ras/pT3) as a control (Figure 3A and 3B). All mice were sacrificed when they became moribund or 8 weeks post injection (end of the experiments).

As previously reported, all c-Myc/pT3 injected mice died of lethal burden of liver tumors by 6.5 weeks post injection [20]. Histological analysis of liver lesions revealed that tumors consisted of cells of small size with a high nuclear/cytoplasm ratio, consistent with their hepatoblastoma nature [20]. In addition, all AKT/Ras/pT3 injected mice died by 8 weeks post injection. Tumors in AKT/Ras/pT3 mice were predominantly HCC, with a small percentage (~10%) of intrahepatic cholangiocarcinomas, in agreement with previous findings [21].

Among all the miRNAs tested, overexpression of mir-378 had no or very limited activity in inhibiting c-Myc or AKT/Ras hepatocarcinogenesis in mice (Table 1 and Supplementary Figure 3A–3D). Indeed, when miR-378 was overexpressed in combination with c-Myc, all c-Myc/miR-378 injected mice developed palpable abdominal mass and became moribund by 6 weeks post injection. Grossly, multiple tumor nodules occupied the whole liver parenchyma. Histological evaluation showed that c-Myc/miR-378 liver tumors were indistinguishable from those developed in c-Myc/pT3 mice. Similarly, all AKT/Ras/miR-378 injected mice developed lethal burden of liver tumors by 6.5 weeks post injection, and histologically AKT/Ras/miR-378 tumors were identical to AKT/Ras/pT3 tumors (Supplementary Figure 3A–3D).

**Table 1:** Summary of tumor suppressor activity of miRNAs in c-Myc or AKT/Ras induced hepatocarcinogenesis (−) no tumor suppressor activity; (+) weak tumor suppressor activity; (++) moderate tumor suppressor activity; (+++): strong tumor suppressor activity.

miRNA	Oncogene	Growth Inhibition
miR-101	c-Myc	+++
	AKT/Ras	+++
miR-107	c-Myc	+
	AKT/Ras	++
miR-122	c-Myc	++
	AKT/Ras	++
miR-29	c-Myc	++
	AKT/Ras	+
miR-365	c-Myc	++
	AKT/Ras	++
miR-375	c-Myc	+
	AKT/Ras	+++
miR-378	c-Myc	−
	AKT/Ras	−
miR-802	c-Myc	++
	AKT/Ras	−

Taken together, the present results indicate that miR-378 does not possess tumor suppressor activity on c-Myc and AKT/Ras induced hepatocarcinogenesis in mice.

### Weak to moderate tumor suppressor potential of miR-107, miR-122, miR-29, miR-365, and miR-802 in c-Myc and AKT/Ras driven liver tumor development

Overexpression of miR-107 slightly delayed c-Myc induced liver tumor formation (Supplementary Figure 4A and 4B). Indeed, 2 out of 4 c-Myc/miR-107 mice developed high tumor burden by eight weeks post injection. On the other hand, miR-107 showed moderate suppressor activity against AKT/Ras induced hepatocarcinogenesis: indeed, none of AKT/Ras/miR-107 injected mice developed enlarged abdomen, and all mice were alive by eight weeks post injection. Grossly, numerous liver tumor nodules were present throughout the liver in these mice, although the tumor burden was significantly decreased compared with that of AKT/Ras/pT3 control mice (Supplementary Figure 4C and 4D).

Overexpression of miR-122 delayed c-Myc induced hepatocarcinogenesis, as two of nine c-Myc/miR-122 injected mice developed high tumor burden 8 weeks post injection (Supplementary Figure 5A and 5B). The tumor suppressor activity of miR-122 against AKT/Ras induced liver tumor was even more pronounced, as all of the AKT/Ras/miR-122 injected mice appeared to be healthy 8 weeks post injection. Macroscopically, livers from AKT/Ras/miR-122 injected mice were pale, but no tumor nodules were detected. Histologically, AKT/Ras/miR-122 livers showed the presence of clusters of lipid-rich preneoplastic hepatocytes occupying most of the liver parenchyma, whereas no frankly malignant lesions were identified (Supplementary Figure 5C and 5D).

Overexpression of miR-29 slightly delayed AKT/Ras and c-Myc driven liver tumor formation. However, in both models, significant liver tumor burden was observed 8 weeks post injection (Supplementary Figure 6A–6D). Similar results were obtained when co-expressing miR-365 with c-Myc or AKT/Ras (Supplementary Figure 7A–7D).

Overexpression of miR-802 did not affect AKT/Ras induced liver tumor formation, with all AKT/Ras/miR-802 injected mice being euthanized due to high tumor burden by 8 weeks post injection (Supplementary Figure 8C and 8D). On the other hand, none of c-Myc/miR-802 injected mice showed enlarged liver by 8 weeks post injection. Histologically, all c-Myc/miR-802 injected mice developed liver tumors, although the tumor burden was much lower than that of c-Myc/pT3 mice. Histologically, tumors developed in c-Myc/miR-802 mice were hepatoblastomas, identical to c-Myc/pT3 tumors (Supplementary Figure 8A and 8B).

In summary, the present results indicate that miR-107, miR-122, miR-29, miR-365, and miR-802 possess weak to moderate tumor suppressive properties, as none of them is able to completely prevent oncogene driven liver tumor development in mice.

### miR-375 strongly inhibits AKT/Ras hepatocarcinogenesis but not c-Myc induced liver tumor formation

**Figure 4:**
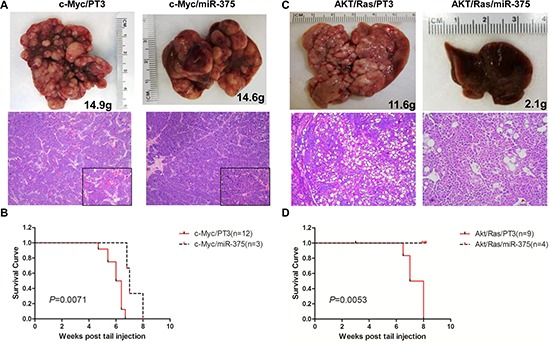
Overexpression of miR-375 strongly inhibits AKT/Ras but not c-Myc induced liver tumor formation in mice **(A)** Macroscopic (upper panel) and microscopic (lower panel) appearance of livers from c-Myc/pT3 mice and c-Myc/miR-375 mice stained with H&E (100X), insets (400X). **(B)** Kaplan Meier survival curve of c-Myc/pT3 and c-Myc/miR-375 mouse cohort. **(C)** Macroscopic (upper panel) and microscopic (lower panel) appearance of livers from AKT/Ras/PT3 mice and AKT/Ras/miR-375 mice stained with H&E (100X). **(D)** Kaplan Meier survival curve of AKT/Ras/pT3 and AKT/Ras/miR-375 mouse cohort.

Overexpression of miR-375 slightly delayed c-Myc induced liver tumor formation (Figure 4A and 4B). By 8 weeks post injection, all c-Myc/miR-375 injected mice succumbed due to the high tumor burden (Figure 4B). In striking contrast, miR-375 exhibited a strong tumor suppressor activity against AKT/Ras driven tumor development (Figure 4C and 4D). Indeed, none of the AKT/Ras/miR-375 injected mice showed any sign of tumor development 8 weeks post injection. Macroscopically, liver appeared to be normal (Figure 4C), while microscopically few, small clusters of lipid-rich preneoplastic hepatocytes were detected. Thus, the present findings support a strong tumor suppressive role of miR-375 against AKT/Ras driven hepatocarcinogenesis and a limited antineoplastic activity toward c-Myc induced liver tumor formation.

### Overexpression of miR-101 completely suppresses liver tumor formation induced by c-Myc and AKT/Ras oncogenes

**Figure 5:**
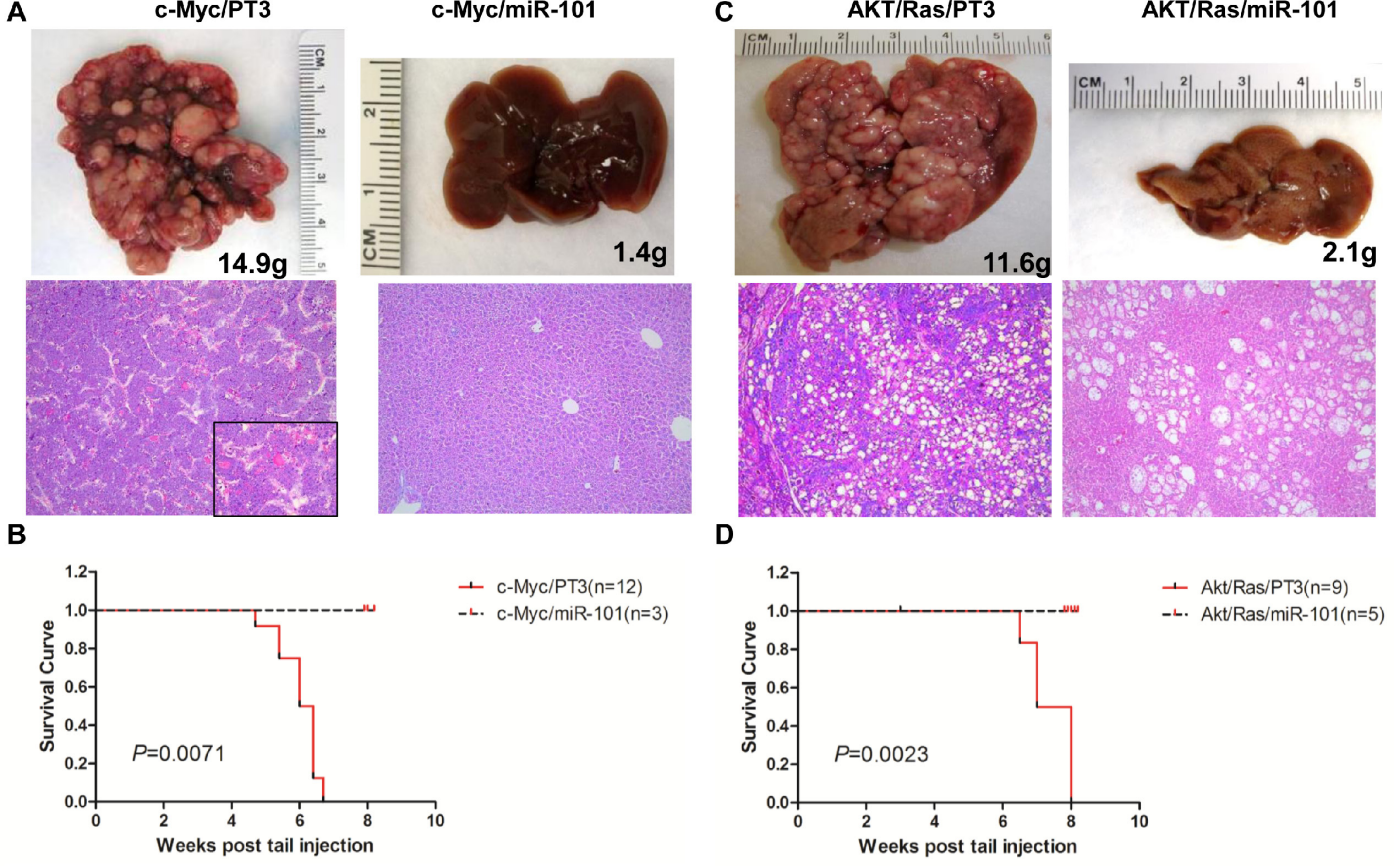
Overexpression of miR-101 efficiently inhibits c-Myc and AKT/Ras induced liver tumor development **(A)** Macroscopic (upper panel) and microscopic (lower panel) appearance of livers from c-Myc/pT3 mice and c-Myc/miR-101 mice stained with H&E (100X), insets (400 X). **(B)** Kaplan Meier survival curve of c-Myc/pT3 and c-Myc/miR-101 mouse cohort. **(C)** Macroscopic (upper panel) and microscopic (lower panel) appearance of livers from AKT/Ras/PT3 mice and AKT/Ras/miR-101 mice stained with H&E (100X). **(D)** Kaplan Meier survival curve of AKT/Ras/pT3 and AKT/Ras/miR-101 mouse cohort.

Different from all the other tumor suppressor miRNAs tested, overexpression of miR-101 completely prevented both c-Myc and AKT/Ras driven liver tumor formation in mice. Specifically, all c-Myc/miR-101 injected mice appear to be healthy 8 weeks post injection (Figure 5A and 5B). Upon dissection, no liver tumor nodules were identified, and livers of c-Myc/miR-101 injected mice appeared to be completely normal histologically (Figure 5A). Similarly, none of the AKT/Ras/miR-101 injected mice showed any sign of tumor development at the same time point (Figure 5C and 5D). Livers from AKT/Ras/miR-101 injected mice were slightly pale, and clusters of lipid-rich preneoplastic hepatocytes were detected at the microscopic level (Figure 5A).

In summary, the present data indicate that miR-101 possesses a broad anti-tumor activity against mouse liver tumors induced by different oncogenes.

## DISCUSSION

HCC is a highly heterogeneous disease, with distinct molecular mechanisms playing a pathogenetic role in different tumor subgroups. Thus, it is conceivable that molecular-based therapeutic approaches might be effective only in certain patients’ subsets.

In the present investigation, we assessed whether liver tumors induced by the overexpression of different oncogenes display distinct miRNA expression profiles. Furthermore, we determined the anti-oncogenic potential of selected miRNAs on hepatocarcinogenesis driven by different oncogenes. Our results indicate that distinct miRNA expression profiles characterize liver tumors induced by c-Myc and AKT/Ras oncogenes. In addition, the distinct expression signatures of c-Myc and AKT/Ras tumors exhibit distinct power in predicting the prognosis of human HCC patients.

To examine the tumor suppressor activity of selected miRNAs, we applied the hydrodynamic transfection approach and co-expressed individual miRNA with c-Myc or AKT/Ras oncogenes. Hydrodynamic transfection is an innovative approach that combines hydrodynamic injection [25] with sleeping beauty mediated somatic integration [26] for long term and stable gene expression in mouse hepatocytes. Hydrodynamic transfection has become increasingly popular in the liver cancer research community and is now widely used for *in vivo* modeling of liver tumor development, to elucidate the biochemical crosstalk between signaling pathways as well as to test novel therapeutic agents for the treatment of liver cancer [27]. However, no study has been carried out using hydrodynamic transfection to investigate the functional contribution of miRNA during liver cancer development to date. In the present study, we evaluated the tumor suppressing activity of 8 distinct miRNAs that were found to be downregulated in mouse and human liver tumors. Our findings demonstrate a variable tumor suppressor activity of the eight miRNAs tested and suggest that hydrodynamic transfection could be applied as a reliable and cost-effective approach to study the functional role of miRNAs in hepatocarcinogenesis. While the present study focused on putative tumor suppressor miRNAs, a similar approach can be applied to study candidate oncogenic miRNAs as well.

As concerns c-Myc and AKT/Ras driven hepatocarcinogenesis, our results suggest that different miRNAs might play distinct functional roles in tumors driven by different oncogenic events. In other words, one miRNA might possess strong tumor suppressive properties against hepatocarcinogenesis driven by a certain oncogene, while having no or very limited anti-neoplastic activity in a tumor induced by a different oncogenic alteration. In agreement with the latter hypothesis, we found that miR-375 has limited tumor suppressor activity against c-Myc driven hepatocarcinogenesis, whereas it strongly inhibits AKT/Ras dependent liver tumor formation. These findings suggest that genes targeted by miR-375 may have critical roles in AKT/Ras but not in c-Myc driven hepatocarcinogenesis. miR-375 was first identified as a pancreatic islet-specific miRNA involved in insulin secretion [28]. Recently, it has been shown that the expression of miR-375 is significantly downregulated in multiple tumor types [29], including HCC [30]. miR-375 functions via targeting multiple genes involved in tumor development, including Yap [30], PDK1 [31], 14–3-3-ζ [31] and SHOX2 [32]. The mechanisms underlying the tumor suppressor activity of miR-375 on AKT/Ras dependent hepatocarcinogenesis remain to be defined.

Liver cancer is one of the most frequent types of tumor worldwide. The treatment of liver cancer can be challenging largely due to the fact most of the liver tumor patients have underlying liver diseases, including fibrosis and cirrhosis. Thus, prevention of HCC might be more effective than treatment of HCC. Since most HCCs develop in the presence of a long lasting predisposing condition, such as HBV or HCV chronic infection, alcohol consumption, or non-alcoholic steatohepatitis, chemoprevention of HCC might be quite effective. One of the major challenges of chemoprevention is that HCC is a heterogeneous disease, and HCCs may arise from different genetic alterations. miRNAs hold great promise as chemopreventive agents, as they simultaneously target multiple genes, and thus might possess a broad anti-neoplastic activity toward tumors driven by distinct genetic alterations. In accordance with the latter hypothesis, in the present study we showed that miR-101 effectively prevents liver tumor development initiated by c-Myc and AKT/Ras oncogenes, providing strong evidence that miR-101 may be an ideal candidate for miRNA-based chemoprevention of HCC. Despite the latter encouraging results, we acknowledge that our current study is limited as we only investigated the miR-101 in two oncogene-induced liver tumor murine models. Clearly, hepatocarcinogenesis is a multi-step and complex process, involving many biological events and signaling pathways. Thus, additional investigations in other murine liver cancer models are necessary to further establish the tumor suppressor role of miR-101 in HCC [33]. For instance, transgenic mice overexpressing viral genes, such as HBX [34] or HCV Core [35] transgenic mice, might be used to test whether miR-101 inhibits hepatitis virus-driven HCC development. Furthermore, models of chemically-induced HCC, such as the diethylnitrosamine (DENA) model, might be helpful to evaluate the tumor suppressor activity of miR-101 in the context of liver injury and inflammation.

miR-101 has been shown to be downregulated in multiple tumor types, including HCC [36]. miR-101 targets many genes associated with tumorigenesis, such as Mcl-1 [36], EZH2 [37] and STMN1 [38]. In accordance with the latter findings, we found that Mcl-1, EZH2, and STMN1 genes were significantly down-regulated following miR-101 overexpression in c-Myc [39] and AKT/Ras [21] mouse HCC cell lines (Supplementary Figure 9), suggesting that these genes are miR-101 targets in mouse liver tumors. Consistent with our observation, overexpression of miR-101 is able to inhibit the growth of human HCC cell lines with different genetic background, including HepG2 [36], QGY-7703 [36], BEL-7402 [37], Hep3B [40], and Huh7 [40] cells. Previous studies showed that Mcl-1, an antiapoptotic member of the Bcl-2 family, is a *bona fide* target of miR-101 in HCC [36]. Of note, preliminary results from our group indicate that co-expression of miR-101 and a Mcl-1 form lacking the 3′ untranslated region (thus impeding the binding of Mcl-1 to miR-101) via hydrodynamic transfection leads to the impairment of miR-101 tumor suppressor activity in AKT/Ras and c-Myc mice. Indeed, AKT/Ras/miR-101/Mcl1 and c-Myc/miR-101/Mcl-1 injected mice developed large tumors within 6 weeks post injection, whereas AKT/Ras/miR-101/pT3 and c-Myc/miR-101/PT3 mice were completely healthy at the same time point (Supplementary Figure 10A and 10B). These data, together with the downregulation of Mcl-1 in c-Myc and AKT/Ras cell lines, suggest that Mcl-1 may be a crucial target of miR-101 in AKT/Ras and c-Myc hepatocarcinogenesis. Nonetheless, additional investigations are needed to identify crucial miR-101 targets in liver tumor development.

In summary, we have shown that distinct miRNAs profiles characterize liver tumors induced by distinct oncogenes. We have also identified some miRNAs whose overexpression is able to either delay or abolish tumor development in aggressive mouse models of liver cancer. The elevated anti-tumor activity of some of the miRNAs identified together with the absence of major side effects in the mouse suggest that *in vivo* manipulation of miRNAs might represent an innovative and effective therapeutic approach against human HCC.

## MATERIALS AND METHODS

### miRNA profiling in livers of c-Myc and AKT/Ras mice

To identify the miRNAs that are differentially expressed between c-Myc or AKT/Ras and wild-type mice, we used NanoString nCounter miRNA (NanoString Technologies, Seattle, WA) assay to perform miRNA profiling in livers of wild-type, c-Myc and AKT/Ras mice. Total RNA was extracted from frozen liver tissue using miRNeasy Kit (Qiagen, Valencia, CA) according to the manufacturer's protocol. Only RNA samples with good RNA quality as confirmed with the Agilent 2100 Bioanalyzer (Agilent Technologies, Santa Clara, CA) were included for the array study. Total RNA was used for the nCounter microRNA platform. All sample preparation and hybridization were performed according to the manufacturer's instructions. All hybridization reactions were incubated at 65°C for a minimum of 12 hours. Hybridized probes were purified and counted on the nCounter Prep Station and Digital Analyzer (NanoString Technologies) following the manufacturer's instructions. For each assay a high-density scan was performed. For platform validation using synthetic oligonucleotides, NanoString nCounter microRNA raw data were normalized for lane-to-lane variation with a dilution series of six spike-in positive controls. The sum of the six positive controls for a given lane was divided by the average sum across lanes to yield a normalization factor, which was then multiplied by the raw counts in each lane to give normalized values.

### Microarray analysis

Normalized micorRNA array data was obtained and used for the analysis. Unsupervised clustering was performed using Genesis 1.7.6 with Euclidean distance. The comparison of miRNA profiles between c-Myc and AKT/Ras induced tumors was performed using un-paired student *t*-test. *P*-values < 0.05 were considered to be significant.To identify c-Myc and AKT/Ras signatures, we performed unpaired student *t*-test of c-Myc induced tumors (or AKT/Ras tumors) vs. normal livers. miRNAs with *p*-value < 0.001 and intensity fold change ≥ 2.0 (on either tumor vs. non-tumor or non-tumor vs. tumor) were included in the signatures.

A previously described human HCC clinical dataset (*n* = 241) with available miRNA profiles [22–24] was used in this study to compare mouse liver tumor miRNA profile with human HCC miRNA profile. Human HCC miRNA profiles were assessed in the single channel miRNA microarray platform (V 2.0, GEO accession number: GSE6857) [22–24]. As concerns the human cohort, we have used tissue specimens from 241 HCC patients who had undergone radical liver tumor resection between 1999 and 2003 at the Fudan University in Shanghai, China. Most of the patients were men (87%), were long-term carriers of hepatitis B virus (HBV) (93%), had an elevated serum level of alpha-fetoprotein (68%), had a single-nodule disease (89%), and a tumor size larger than 3 cm (63%). The median survival time was more than 60 months, and the range of survival time was 2–67 months after tumor resection. Among the 241 liver cancer cases, 227 cases had available clinical survival information. Subgroups of HCC patients were predicted by the c-Myc signature or AKT/Ras signature using Genesis 1.7.6 with hierarchical clustering analysis. In detail, the data matrix, including the expression level of genes from signature and patients ID, was first normalized in Genesis. Further, the Euclidean distance clustering with the complete linkage agglomeration rule was performed to predict the patient subgroups. Kaplan–Meier survival analysis was used to compare patients’ survival based on prediction results using PrismGraph V6.0 (San Diego, CA), and the statistical *P* value was generated by the Cox–Mantel log-rank test.

### Constructs and reagents

A region of ~400 bp region encompassing each miRNA was amplified from mouse genomic DNA, and cloned into pT3-EF1α vector for hydrodynamic transfection. The primer sequences for amplifying each miRNA are shown in Supplementary. Table 1D. c-Myc/pT3-EF1α [20], myr-AKT/pT3-EF1α [41], NRasV12/pT2-CAGGS [26], and pCMV/SB [26] have been described previously. All the plasmids were purified using the Endotoxin free Maxi prep kit (Sigma, St. Louis, MO).

### Hydrodynamic transfection

Wild-type FVB/N mice were obtained from Charles River (Wilmington, MA) and were used at 6 to 8 weeks of age. Hydrodynamic injections were performed as described [27]. In brief, to determine the tumor suppressing activity of miRNAs, mice were hydrodynamically injected either with 4 μg c-Myc/pT3EF1α, 10 μg miRNA/pT3EF1α together with 0.56 μg pCMV/SB or with 4 μg myr-AKT/pT3EF1α, 4 μg NRasV12/pT2-CAGGS, 10 μg miRNA/pT3EF1α together with 0.72 μg pCMV/SB. Control mice were injected with 4 μg c-Myc/pT3EF1α, 10 μg pT3EF1α together with 0.56 μg pCMV/SB; or 4 μg myr-AKT/pT3EF1α, 4 μg NRasV12/pT2-CAGGS, pT3EF1α together with 0.72 μg pCMV/SB. Mice were housed, fed, and monitored in accordance with protocols approved by the Committee for Animal Research at the University of California, San Francisco.

### Histology

The mice were euthanized, liver tissues were collected, fixed in 4% paraformaldehyde, and embedded in paraffin. Slides were sectioned and stained with H&E for histological evaluation.

## SUPPLEMENTARY FIGURES AND TABLES




