# Supplementary material for: Distinct anti-oncogenic effect of various microRNAs in different mouse models of liver cancer
Source: Oncotarget. 2015 Feb 9;6(9):6977–88. doi: 10.18632/oncotarget.3166 (PMC4466663; doi:10.18632/oncotarget.3166)
Supplement: Supplementary file 1 [file oncotarget-06-6977-s001.pdf]

SUPPLEMENTARY FIGURES AND TABLES

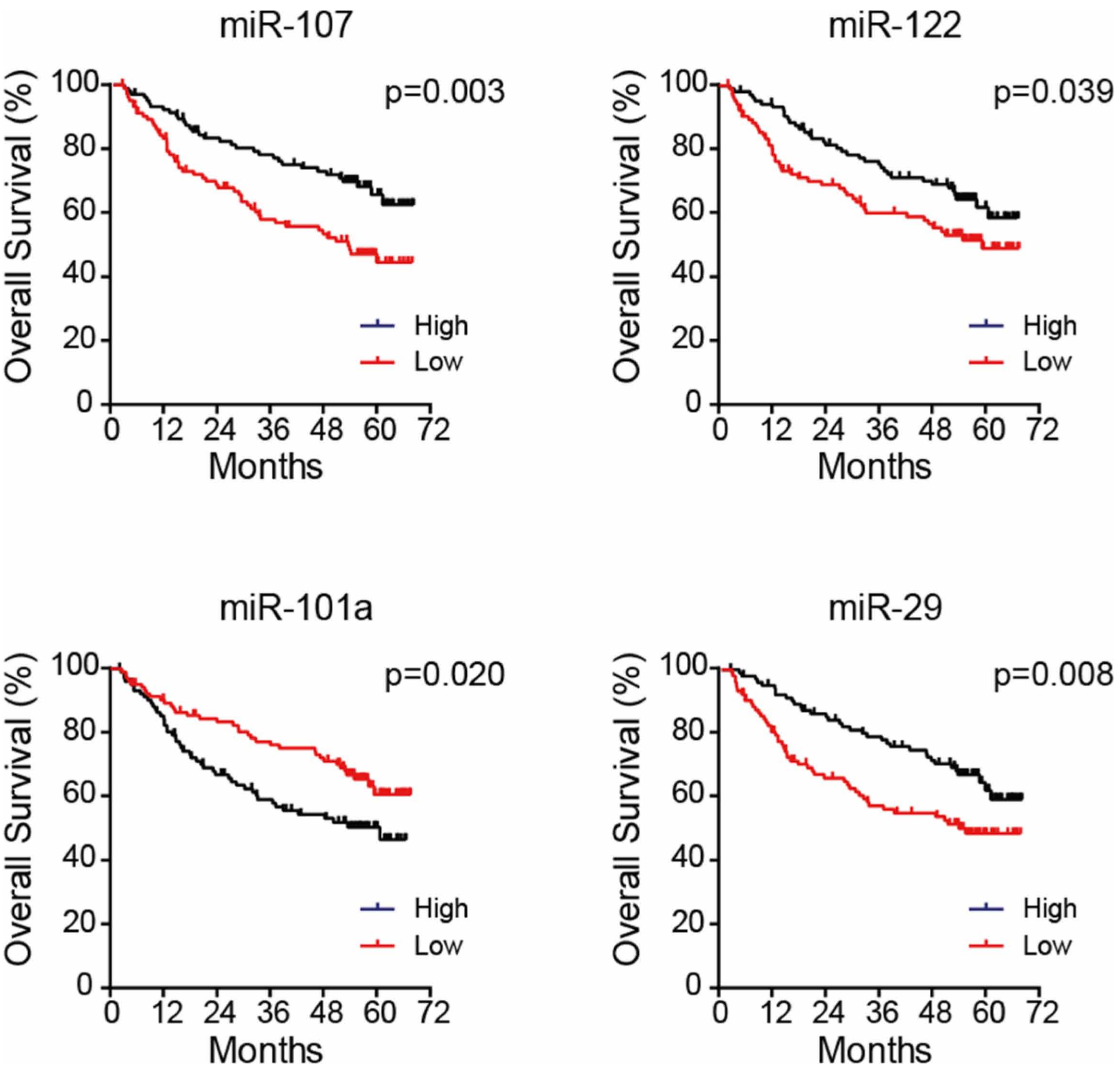

Supplementary Figure 1: Kaplan-Meyer curves showing that the expression of miR-107, miR-122, miR-101 and miR-29 in liver tumors is significantly associated with the patients' outcome.

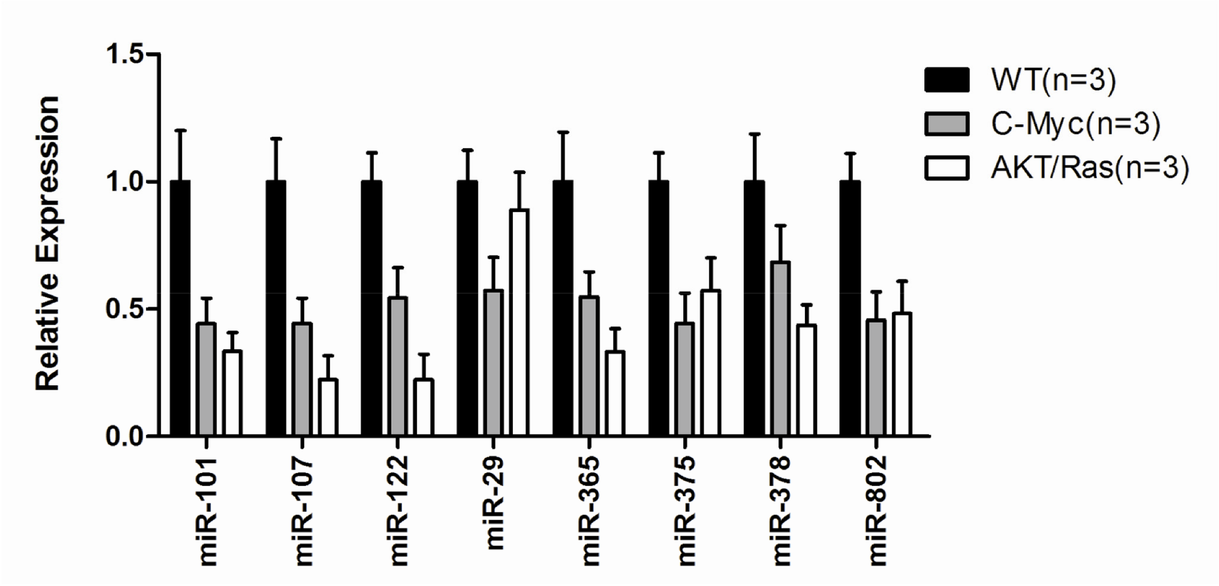

Supplementary Figure 2: Quantitative real-time RT-PCR data showing that miR-101, miR-107, mir-122, miR-29, miR-365, miR-375, miR-378, and miR-802 are all downregulated in c-Myc and AKT/Ras liver tumor tissues.

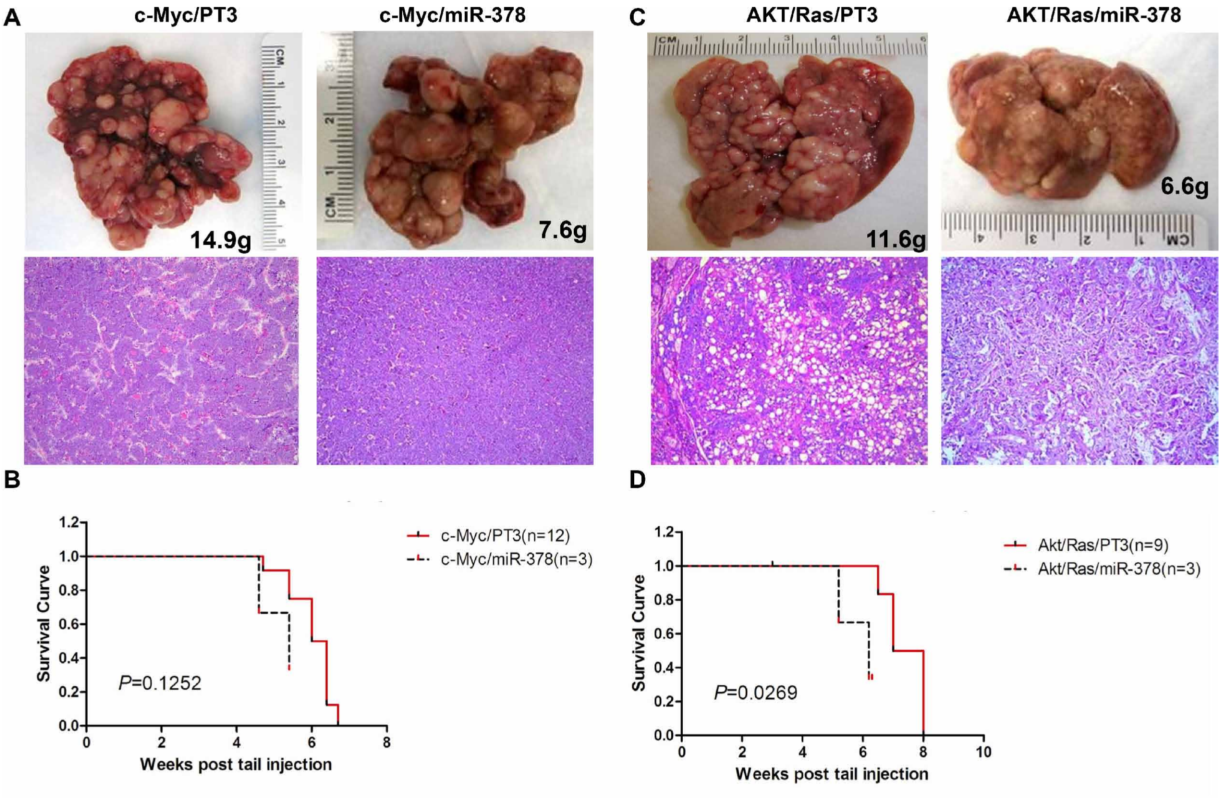

Supplementary Figure 3: Lack of tumor suppressor activity of miR-378 in c-Myc and AKT/Ras induced liver tumor formation in mice. **(A)** AMacroscopic (upper panel) and microscopic (lower panel) appearance of livers from c-Myc/PT3 mice and c-Myc/miR-378 mice stained with H&E (100X). **(B)** Kaplan Meier curve showing that co-expressing miR-378 does not inhibit c-Myc induced liver tumor development. **(C)** Macroscopic (upper panel) and microscopic (lower panel) appearance of livers from AKT/Ras/PT3 mice (control) and AKT/Ras/miR-378 mice stained with H&E (100X). **(D)** Kaplan Meier curve showing that co-expressing miR-378 does not inhibit AKT/Ras induced liver tumor formation in mice.

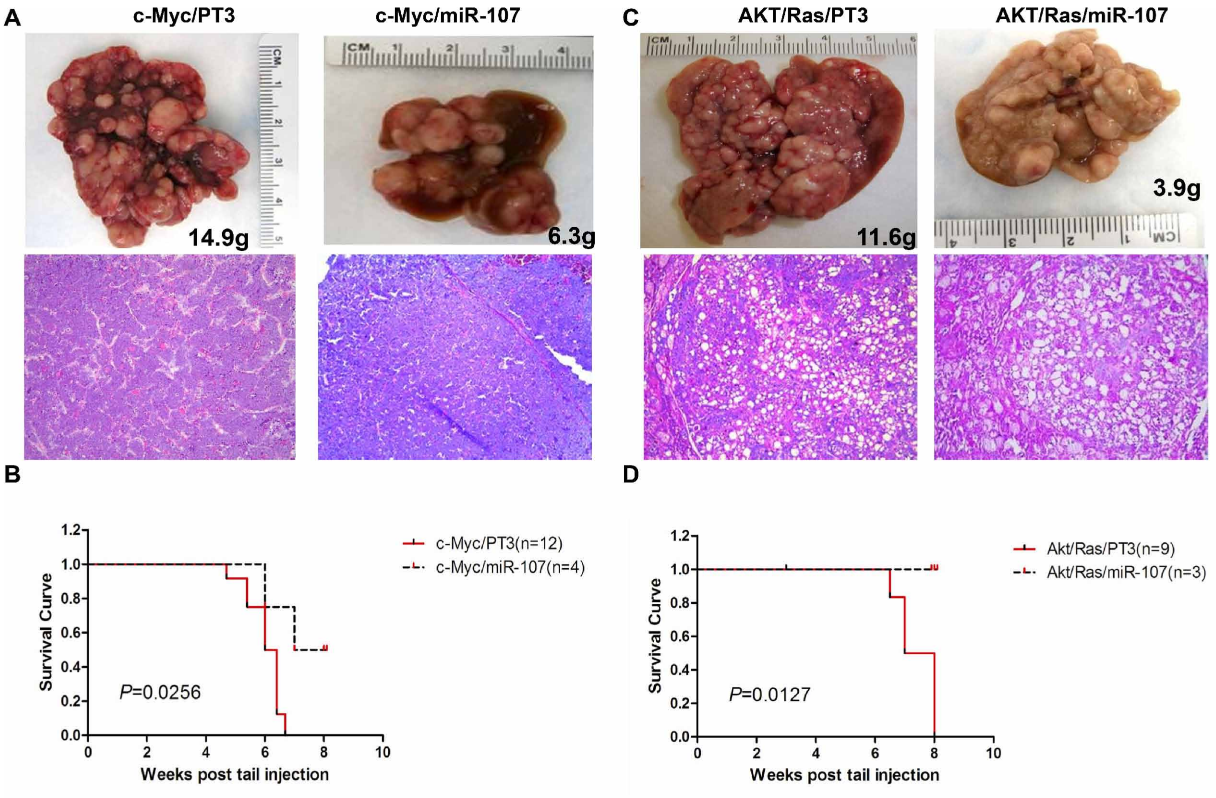

Supplementary Figure 4: Tumor suppressor activity of miR-107 in c-Myc or AKT/Ras induced liver tumor formation. **(A)** Macroscopic (upper panel) and microscopic (lower panel) appearance of livers from c-Myc/PT3 mice and c-Myc/miR-107 mice stained with H&E (100X). **(B)** Kaplan Meier curve shows that overexpression of miR-107 slightly delays c-Myc induced liver tumor formation. **(C)** Macroscopic (upper panel) and microscopic (lower panel) appearance of livers from AKT/Ras/PT3 mice and AKT/Ras/miR-107 mice stained with H&E (100X). **(D)** Kaplan Meier curve shows that co-expression of miR-107 strongly inhibits AKT/Ras induced liver tumor formation in mice.

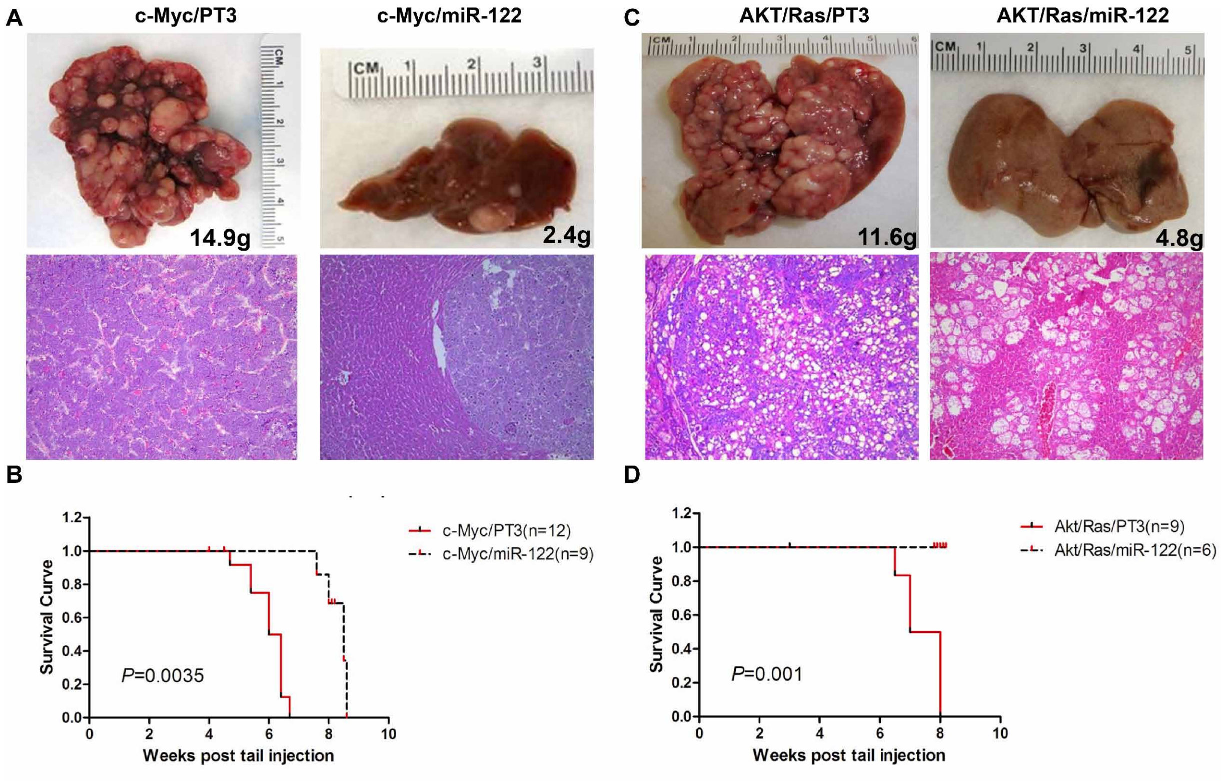

Supplementary Figure 5: Tumor suppressor activity of miR-122 in c-Myc or AKT/Ras induced liver tumor formation. (A) Macroscopic (upper panel) and microscopic (lower panel) appearance of livers from c-Myc/PT3 mice and c-Myc/miR-122 mice stained with H&E (100X). **(B)** Kaplan Meier curve shows that overexpression of miR-122 delays c-Myc induced liver tumor formation. **(C)** Macroscopic (upper panel) and microscopic (lower panel) appearance of livers from AKT/Ras/PT3 mice and AKT/Ras/miR-122 mice stained with H&E (100X). **(D)** Kaplan Meier curve showes that co-expressing miR-122 strongly inhibits AKT/Ras induced liver tumor formation in mice.

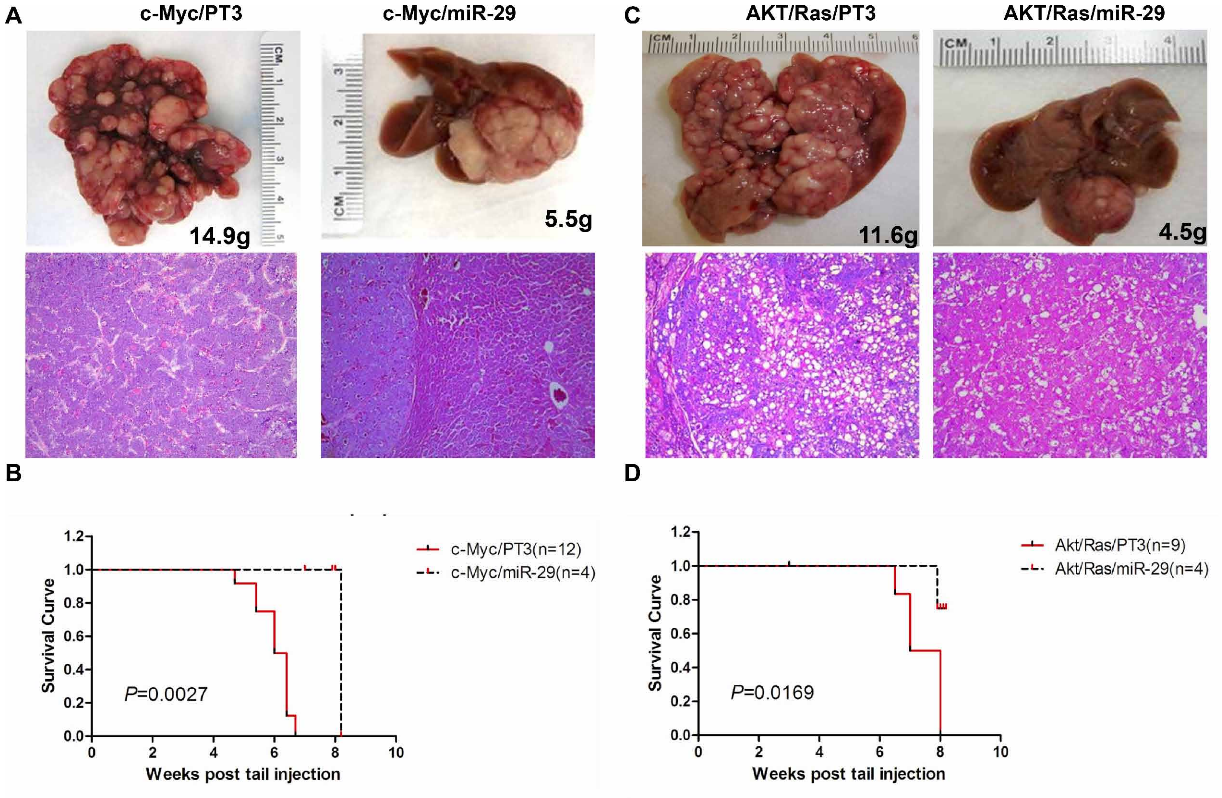

Supplementary Figure 6: Tumor suppressor activity of miR-29 in c-Myc or AKT/Ras induced liver tumor formation. (A) Macroscopic (upper panel) and microscopic (lower panel) appearance of livers from from c-Myc/PT3 mice and c-Myc/miR-29 mice stained with H&E (100X). **(B)** Kaplan Meier curve showing that overexpression of miR-29 slightly delays c-Myc induced liver tumor formation. **(C)** Macroscopic (upper panel) and microscopic (lower panel) appearance of livers from from AKT/Ras/PT3 mice and AKT/Ras/miR-29 mice stained with H&E (100X). **(D)** Kaplan Meier curve shows that co-expressing miR-29 slightly inhibits AKT/Ras induced liver tumor formation in mice.

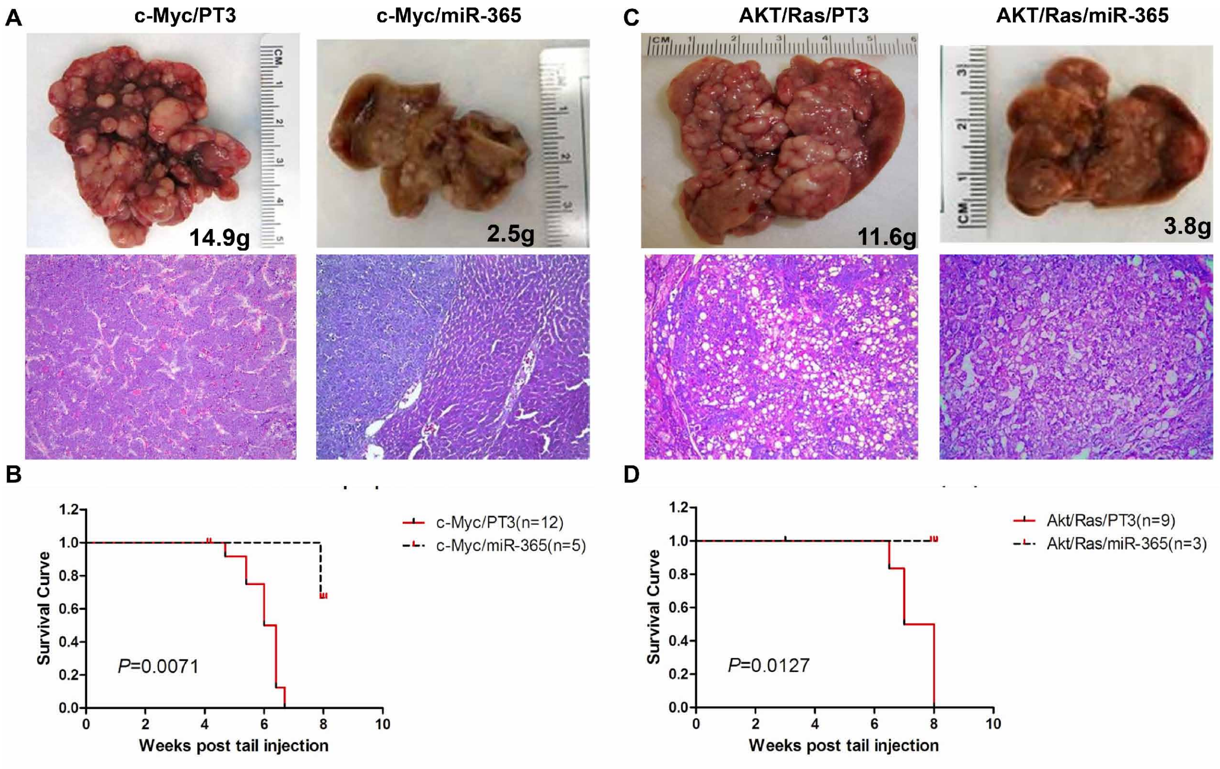

Supplementary Figure 7: Tumor suppressor activity of miR-365 in c-Myc or AKT/Ras induced liver tumor formation. **(A)** Macroscopic (upper panel) and microscopic (lower panel) appearance of livers from from c-Myc/PT3 mice and c-Myc/miR-365 mice stained with H&E (100X). **(B)** Kaplan Meier curve shows that overexpression of miR-365 slightly delays c-Myc induced liver tumor formation. **(C)** Macroscopic (upper panel) and microscopic (lower panel) appearance of livers from from AKT/Ras/PT3 mice and AKT/Ras/miR-365 mice stained with H&E (100X). **(D)** Kaplan Meier curve shows that co-expressing miR-365 inhibits AKT/Ras induced liver tumor formation in mice.

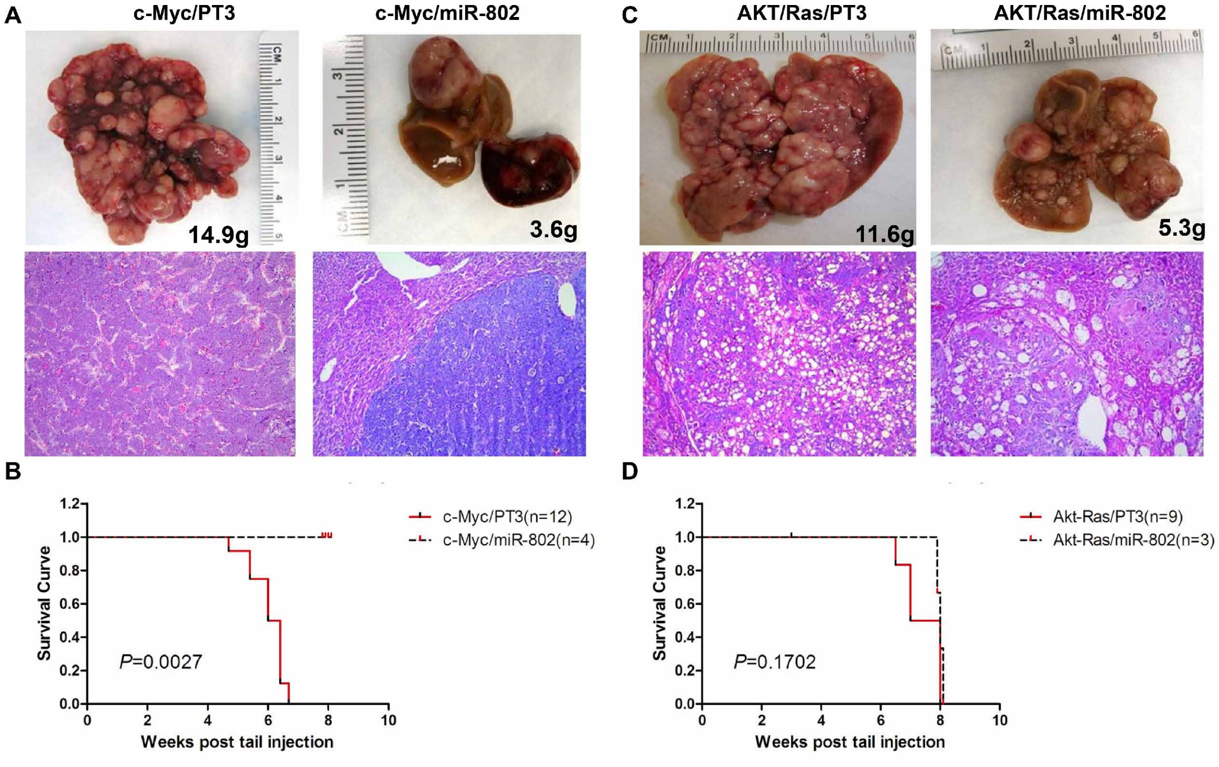

Supplementary Figure 8: Tumor suppressor activity of miR-802 in c-Myc or AKT/Ras induced liver tumor formation. (A) Macroscopic (upper panel) and microscopic (lower panel) appearance of livers from from c-Myc/PT3 mice and c-Myc/miR-802 mice stained with H&E (100X). **(B)** Kaplan Meier curve shows that overexpression of miR-802 strongly delays c-Myc induced liver tumor formation. **(C)** Macroscopic (upper panel) and microscopic (lower panel) appearance of livers from from AKT/Ras/PT3 mice and AKT/Ras/miR-802 mice stained with H&E (100X). **(D)** Kaplan Meier curve showing that co-expression of miR-802 does not affect AKT/Ras induced liver tumor formation in mice.

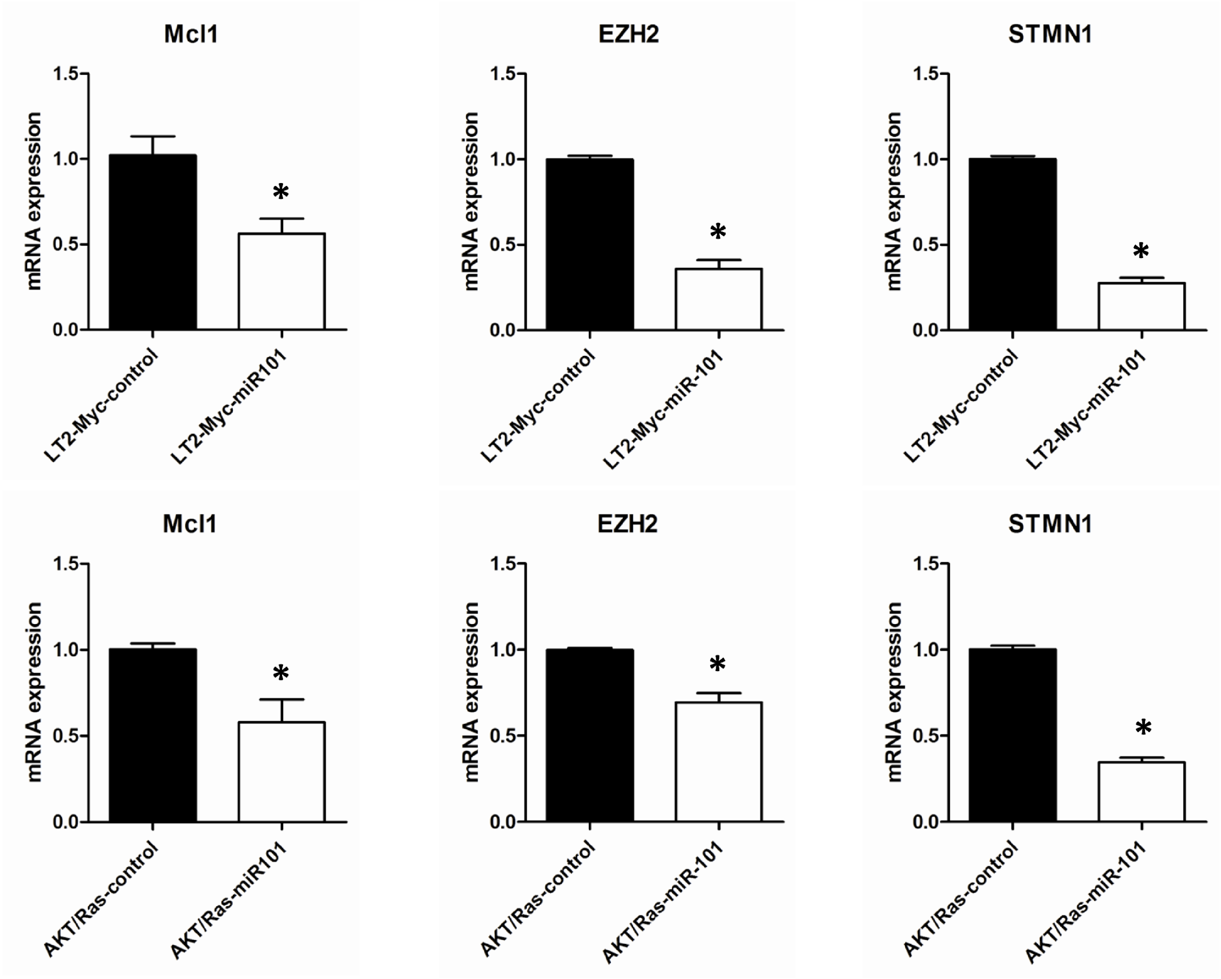

Supplementary Figure 9: Real-time RT-PCR analysis showing that overexpression of miR-101 results in the downregulation of Mcl-1, EZH2 and STMN1 putative target genes in c-Myc (upper panel) and AKT/Ras (lower panel) mouse HCC cell lines (*represents *$P < 0.05$).**

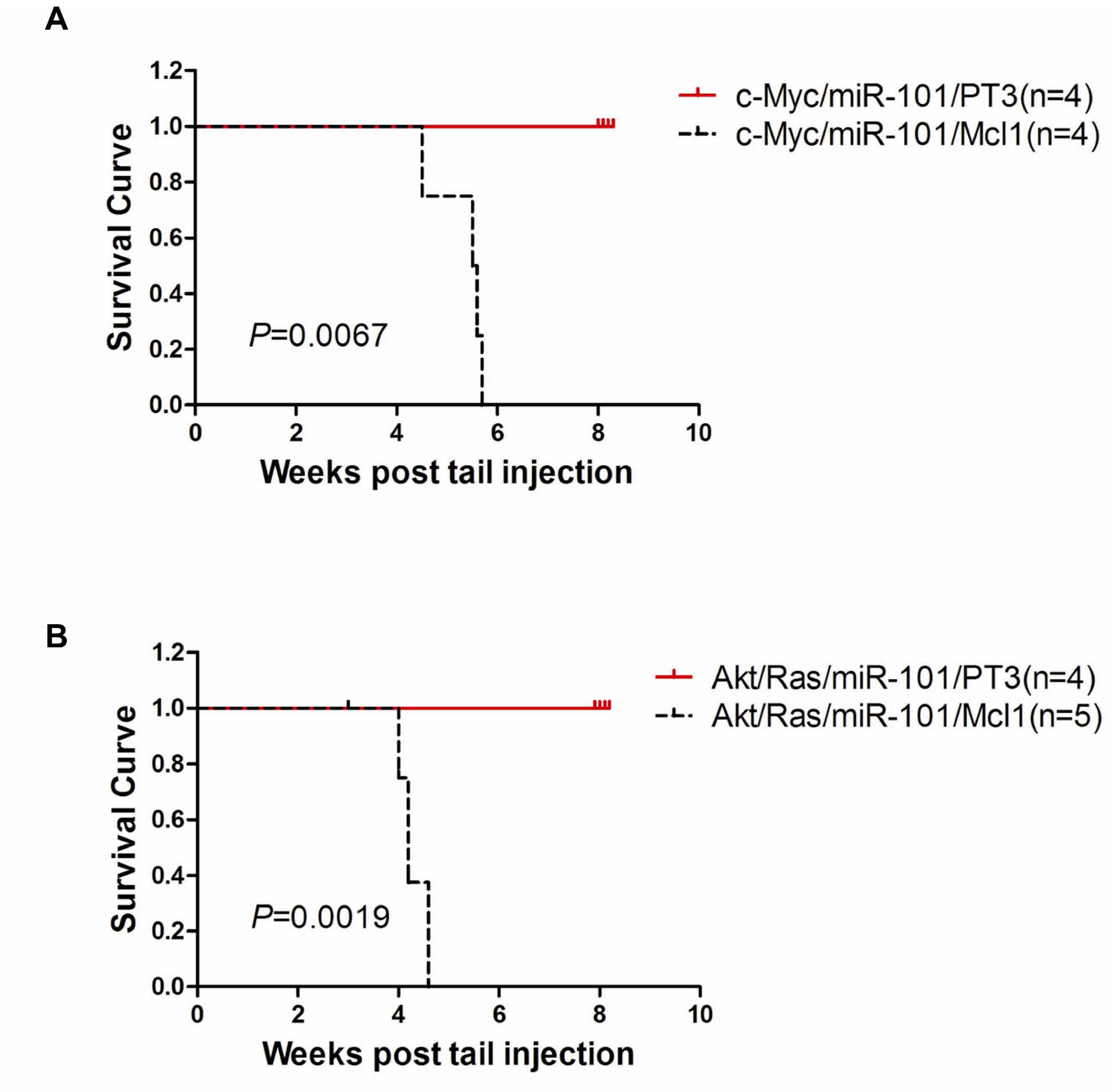

Supplementary Figure 10: Contribution of Mcl-1 in regulating tumor suppressor activity of miR-101 in AKT/Ras and c-Myc mice. The Mcl-1 cDNA clone lacking the 3′ untranslated region, which cannot be targeted by miR-101, was cloned into pT3-EF1a vector. c-Myc/miR-101 plasmids were mixed with Mcl-1 (c-Myc/miR-101/Mcl-1) or empty vector pT3EF1a (c-Myc/miR-101/pT3), and the mixture was delivered into mouse liver via hydrodynamic transfection. Similar experiments were carried out with the AKT/Ras model. Mice were hydrodynamically transfected with AKT/Ras/miR-101/Mcl-1 or AKT/Ras/miR-101/pT3 (empty vector) construct. **(A)** Kaplan Meier curve shows that overexpression of Mcl-1 blocks the inhibitory effect of miR-101 on c-Myc induced liver tumor formation. **(B)** Kaplan Meier curve showing that co-expression of Mcl-1 suppresses the inhibitory effect of miR-101 on AKT/Ras induced liver tumor formation.

Supplementary Table 1A: The global miRNA expression patterns were compared between normal mouse livers and those from liver tumors induced by c-Myc or AKT/Ras oncogenes

Supplementary Table 1B: miRNAs were significantly differently expressed between AKT/Ras tumors and normal liver tissues

AKT/Ras miRNA signature	avaliable in miRNA array of human HCCs	Up/Down regulation (c-myc vs. Control)	Fold change (log 2 value)
mmu-miR-207	yes	Down	–2.33
mmu-miR-192	yes	Down	–1.90
mmu-miR-338-5p	yes	Down	–1.56
mmu-miR-346	yes	Down	–1.33
mmu-miR-1	yes	Down	–1.31
mmu-miR-26a	yes	Down	–1.21
mmu-miR-320	yes	Down	–1.20
mmu-miR-138	yes	Down	–1.19
mmu-miR-99b	yes	Up	1.05
mmu-miR-802	no	Down	–3.09
mmu-miR-1906	no	Down	–2.34
mmu-miR-676	no	Down	–2.01
mmu-miR-1946b	no	Down	–1.97
mmu-miR-717	no	Down	–1.63
mmu-miR-1933-5p	no	Down	–1.59
mmu-miR-1981	no	Down	–1.57
mmu-miR-1959	no	Down	–1.49
mcmv-miR-m108-2-5p.1	no	Down	–1.47
mmu-miR-758	no	Down	–1.45
mmu-miR-1940	no	Down	–1.44
mmu-miR-551b	no	Down	–1.43
mghv-miR-M1-7-3p	no	Down	–1.35
mmu-miR-2136	no	Down	–1.35
mmu-miR-544	no	Down	–1.32
mmu-miR-664	no	Down	–1.32
mmu-miR-673-3p	no	Down	–1.28
mmu-miR-1939	no	Down	–1.03
mmu-miR-376b	no	Up	3.33

Supplementary Table 1C: miRNAs were significantly differently expressed between c-Myc tumors and normal liver tissues

c-Myc miRNA signature	avaliable in miRNA array of human HCCs	Up/Down regulation (c-myc vs. Control)	Fold change (log 2 value)
mmu-miR-148a	yes	Down	–3.96
mmu-miR-29c	yes	Down	–3.94
mmu-miR-22	yes	Down	–3.93
mmu-miR-26b	yes	Down	–2.90
mmu-miR-30a	yes	Down	–2.84
mmu-miR-122	yes	Down	–2.77
mmu-miR-192	yes	Down	–2.73
mmu-miR-30d	yes	Down	–2.60
mmu-miR-296-3p	yes	Down	–2.55
mmu-miR-137	yes	Down	–2.53
mmu-miR-30b	yes	Down	–2.40
mmu-miR-126-5p	yes	Down	–2.38
mmu-miR-139-5p	yes	Down	–2.22
mmu-miR-29a	yes	Down	–2.20
mmu-miR-30e	yes	Down	–2.15
mmu-miR-29b	yes	Down	–2.08
mmu-miR-26a	yes	Down	–2.04
mmu-miR-207	yes	Down	–1.98
mmu-miR-190b	yes	Down	–1.89
mmu-miR-16	yes	Down	–1.78
mmu-miR-346	yes	Down	–1.74
mmu-miR103	yes	Down	–1.66
mmu-miR-152	yes	Down	–1.64
mmu-miR-292-3p	yes	Down	–1.60
mmu-miR-145	yes	Down	–1.55
mmu-miR-1	yes	Down	–1.50
mmu-miR-15a	yes	Down	–1.26
mmu-miR-31	yes	Down	–1.26
mmu-miR-33	yes	Down	–1.25
mmu-miR-211	yes	Down	–1.16
mmu-miR-320	yes	Down	–1.11
mmu-miR-99b	yes	Up	1.33
mmu-let-7i	yes	Up	1.59
mmu-miR-19b	yes	Up	1.59

(*Continued*)

c-Myc miRNA signature	avaliable in miRNA array of human HCCs	Up/Down regulation (c-myc vs. Control)	Fold change (log 2 value)
mmu-miR-125a-5p	yes	Up	2.16
mmu-miR-136	yes	Up	2.23
mmu-miR-106a+mmu-miR-17	yes	Up	2.47
mmu-miR-296-5p	yes	Up	2.61
mmu-miR-20a+mmu-miR-20b	yes	Up	2.70
mmu-miR-19a	yes	Up	2.78
mmu-miR-802	no	Down	–3.61
mmu-miR-1952	no	Down	–2.31
mmu-miR-1940	no	Down	–2.04
mghv-miR-M1-7-3p	no	Down	–1.87
mcmv-miR-m108-2-5p.1	no	Down	–1.86
mghv-miR-M1-5	no	Down	–1.80
mmu-miR-1951	no	Down	–1.78
mmu-miR-676	no	Down	–1.69
mmu-miR-742	no	Down	–1.52
mmu-miR-717	no	Down	–1.51
mmu-miR-741	no	Down	–1.34
mmu-miR-544	no	Down	–1.28
mmu-miR-450b-5p	no	Down	–1.18
mmu-miR-377	no	Up	2.28
mmu-miR-411	no	Up	2.29
mmu-miR-410	no	Up	2.86
mmu-miR-1196	no	Up	3.01
mmu-miR-369-3p	no	Up	3.33
mmu-miR-376c	no	Up	3.79
mmu-miR-495	no	Up	3.87
mmu-miR-376b	no	Up	5.71

Supplementary Table 1D: Primer list of the miRNA used in the experiments

miRNA	Forward primer	Reverse primer
mir-101	5′-CCGGAATTCAATGACCTCTTTTCTT CTGC-3′	5′-ATACTCGAGGAAGAGTGGTGAACACAGGA-3′
mir-107	5′-GCGGAATTCTTCTTTCTAACTGCCATT ACTGCCG-3′	5′-CCGCTCGAGTCTACTTCTCAACCCTTGTTCCTTA-3′
mir-122	5′-TCGGAATTCGTTATTCAAGATCCC GGGGCT -3′	5′-CGGCTCGAGCATACATTACACACAATGGAG-3′
mir-29	5′-GCGGAATTCCATGTAATAAGCCTTCTCT GG-3′	5′-CCGCTCGAGGAAAGTCAAGTTTAAGATAGG-3′
mir-365	5′-CCGGAATTCCAAAGGTTCACGTGTCTT GGA-3′	5′-ATACTCGAGTGAGCACAGCAAGGGAGATG-3′
mir-375	5′-TTGGAATTCACCTGTGGCGTCCGCC ACTG-3′	5′-CGACTCGAGCAGTCTCTGCGACCCATATG-3′
mir-378	5′-CTGGAATTCTAGAAGGCTCAGAG CTGAGC-3′	5′-ATACTCGAGTCTCCGTCAGCACCACACCG-3′
mir-802	5′-GCGGAATTCTGCCTGCTCTGTCT CCCGCAGC-3′	5′-CCGCTCGAGCTTGCAGCTGGAGTTCCATTGGC-3′
